# Distinctive Roles of 5-aza-2′-deoxycytidine in Anterior Agranular Insular and Basolateral Amygdala in Reconsolidation of Aversive Memory Associated with Morphine in Rats

**DOI:** 10.3389/fnbeh.2016.00050

**Published:** 2016-03-15

**Authors:** Peng Liu, JianJun Zhang, Ming Li, Nan Sui

**Affiliations:** ^1^Key Laboratory of Mental Health, Institute of Psychology, Chinese Academy of SciencesBeijing, China; ^2^University of Chinese Academy of SciencesBeijing, China; ^3^Department of Psychology, University of Nebraska–LincolnLincoln, NE, USA

**Keywords:** DNA methylation, 5-aza-2′-deoxycytidine, morphine addiction, conditioned place aversion, insular, amygdala

## Abstract

5-aza-2′-deoxycytidine (5-aza), an inhibitor of DNA methyltransferases (DNMTs), has been implicated in aversive memory and the function of brain region involved in processing emotion. However, little is known about the role of 5-aza in the reconsolidation of opiate withdrawal memory. In the present study, using the morphine-naloxone induced conditioned place aversion (CPA) model in rats, we injected 5-aza into agranular insular (AI), granular insular (GI), basolateral amygdala (BLA) and central amygdala (CeA) immediately after the memory retrieval and tested the behavioral consequences at 24 h, 7 and 14 days after retrieval test. We found that 5-aza injection into AI disrupted the reconsolidation of morphine-associated withdrawal memory, but 5-aza injection into GI had no impact on the reconsolidation. Meanwhile, 5-aza injection into BLA but not CeA attenuated the withdrawal memory trace 14 days later. However, 5-aza administration to rats, in the absence of memory reactivation, had no effect on morphine-associated withdrawal memory. These findings suggest that 5-aza interferes with the reconsolidation of opiate withdrawal memory, and the roles of insular and amygdala in reconsolidation are distinctive.

## Introduction

The reactivation of opiate-withdrawal memories by cues or context previously associated with withdrawal experience motivates drug-seeking behaviors and increases the risk of relapse (Luo et al., 2013). After reactivated, the memories will undergo an unstable state that is called reconsolidation process (Schiller et al., 2010). The disruption of reconsolidation results in a reduction in the motivational properties of stimuli previously associated with aversive outcomes (Hellemans et al., 2006). Actually, interfering with reconsolidation using pharmacological approaches has been shown to erase the memories and prevent relapse (Lee et al., 2006).

Insular cortex and amygdala are the key brain regions for manipulating the drug-related memory reconsolidation (Hellemans et al., 2006; Contreras et al., 2012). The insular cortex is further subdivided into the agranular insular (AI) and granular insular (GI; Cechetto and Saper, 1987). AI and GI are heterogeneous brain regions with distinct functions and neural connections (Moraga-Amaro and Stehberg, 2012). Specifically, the GI receives and encodes primary aversive sensory information (Moraga-Amaro and Stehberg, 2012). AI plays an important role in the reconsolidation of amphetamine-related memory (Contreras et al., 2012). Additionally, studies implicate an indispensable role for the basolateral amygdala (BLA) in the reconsolidation of drug-related withdrawal memories (Hellemans et al., 2006). Our previous studies also showed that infusion of the protein synthesis inhibitor anisomycin (ANI) into the BLA after memory reactivation blocks morphine-associated withdrawal memories reconsolidation (Wu et al., 2014). With regard to the central amygdala (CeA), which is also implicated in drug withdrawal-induced conditioned place aversion (CPA; Watanabe et al., 2003), appears to be selectively involved in mediating the reconsolidation of alcohol-related memories (Barak et al., 2013). Furthermore, there are massive reciprocal connections between the insular cortex and the amygdala (Moraga-Amaro and Stehberg, 2012). The amygdala projection from the insular cortex appears to be organized and targeted all levels of the intra-amygdala connections linking the lateral, basolateral and central nuclei, ultimately turning to motivate behaviors (Shi and Cassell, 1998).

Although much progress has been made in understanding the mechanism of memory reconsolidation, little is known about the mechanism of transcriptional regulation. Emerging evidence suggest that epigenetic mechanisms, including DNA methylation, are essential regulators of memory formation and maintaining (Zovkic et al., 2013). The methylation of cytosine residues on DNA via DNA methyltransferases (DNMTs) negatively regulates transcription (Foulks et al., 2012). Indeed, studies from our laboratory found that DNMTs activity plays a central role in the acquisition and consolidation of drug-related reward memory (Han et al., 2010; Zhang et al., 2014). Similarly, inhibiting the DNMTs impaired the consolidation of contextual fear memories (Monsey et al., 2011). Reconsolidation, a process similar to consolidation, also needed transcriptional regulation, and recent studies found that DNMTs were required for both the reconsolidation of pavlovian fear memories and the reconsolidation-associated neural plasticity in lateral amygdala (Maddox and Schafe, 2011; Maddox et al., 2014), which suggested that DNMTs may also play an important role in the reconsolidation of opiate-withdrawal memories. In the present study, a CPA model was used to investigate region-specific roles of DNMTs inhibitor 5-aza-2′-deoxycytidine (5-aza) in the insular cortex and amygdala in the reconsolidation of morphine-withdrawal memories.

## Materials and Methods

### Subjects

A total of 166 adult male Sprague-Dawley rats (Vital River Laboratory Animal Technology Co. Ltd., Beijing, China) were used, 61 rats were excluded in Pre-C (pre-conditioning), Post-C (post-conditioning) and misplacement of microinjection site, and 105 rats were included in statistic analysis. The rats (weighing 220–250 g, 6–7 weeks) were housed individually in stainless metal mesh cages (25 cm × 22.5 cm × 30 cm) on a 12:12 h light-dark cycle (light on at 08:00) with controlled temperature (22–24°C) and humidity (40–60%). The experimental procedures followed the National Institutes of Health Guide for Care and Use of Laboratory Animals (Publication No. 85-23, revised 1985) and the experimental protocol was approved by Research Ethics Review Board of Institute of Psychology, Chinese Academy of Sciences.

### Surgery

After a week of acclimatization, rats were anesthetized with sodium pentobarbital (75 mg/kg, i.p.) to implant stainless steel guide cannulas (o.d. 0.6 mm, i.d. 0.35 mm, length 11.5 mm for BLA, 11 mm for CeA, 9 mm for AI and GI) bilaterally into AI (1.2 mm anterior to bregma, 5.0 mm lateral to the midline and 6.6 mm ventral to the skull) and GI (1.2 mm anterior to bregma, 5.0 mm lateral to the midline and 5.5 mm ventral to the skull). The coordinates for the amygdala were the following: BLA (2.8 mm posterior to bregma, 4.9 mm lateral to the midline and 8.5 mm ventral to the skull) and CeA (2.6 mm posterior to bregma, 4.2 mm lateral to the midline and 7.9 mm ventral to the skull). All the coordinates were fixed according to the Paxinos and Watson (2007). The guide cannulas were secured with three small screws and dental cement, and a capped stylet was inserted to prevent occlusion. All rats were treated with penicillin to prevent infection (80,000 units) and allowed to recover for 7 days. The rats were handled every other day to reduce handling stress at the time of experiments.

### Drugs and Microinjections

Morphine hydrochloride (5 mg/kg, Qinghai Pharmaceutical, China) and naloxone hydrochloride (0.5 mg/kg, Sigma, Missouri, MO, USA) were dissolved in 0.9% physiological saline and were administered intraperitoneally at volumes of 1.0 ml/kg body weight. The DNA methyltransferase inhibitor 5-aza (Sigma, St. Louis, MO, USA) was diluted in 0.8% acetate to a concentration of 2 μg/μl (Han et al., 2010) in 0.9% sterile saline. The vehicle group received isovolumetric 0.8% acetic acid injections. The day before microinjections, we handled the rats gently, repeatedly removed and inserted all the stylets to avoid stress when microinjection. During the microinjection period, each rat was gently held while the stylet was removed. The drug or vehicle was delivered with a 10 μl Hamilton microsyringe and the injection was given at the rate of 0.25 μl/min over 2 min with the injector cannula remaining in the guide cannula for another 2 min to prevent backflow.

### Apparatus

The apparatus consisted of three-chamber polyvinyl chloride (PVC) boxes. Two large-side chambers (35 cm × 31 cm × 40 cm) were separated by a smaller chamber (14 cm × 31 cm × 40 cm). The two larger chambers differed in their floor textures (bar and grid, respectively) and provided distinct contexts that were paired with morphine or saline injections. Three distinct chambers were separated by manual guillotine doors.

### Behavioral Procedures

To investigate the reconsolidation of morphine-associated withdrawal memory, all rats should be established morphine-associated withdrawal memory firstly. Here, we used an unbiased, counterbalanced CPA model to train rats forming withdrawal memory. The procedure consisted of three phases: Pre-C, conditioning and Post-C. On day 1, baseline preference was assessed by placing the rats in the center compartment of the CPA apparatus. The rats were allowed freely access to all compartments for 15 min. The Pre-C data showed that rats had no preference. The rats that showed a strong preference for either compartment (>540 s) were excluded. Thus, the morphine-naloxone paired compartment was randomly assigned for each rats, the other compartment was paired with saline. On subsequent conditioning days, the rats were trained for eight consecutive days, on days 2, 4, 6 and 8, each rat was injected with saline 4 h after receiving saline injection and immediately confined in its saline-paired compartment for 45 min; on days 3, 5, 7 and 9, the rat was injected with naloxone 4 h after receiving morphine injection to induce enhanced withdrawal and confined to the corresponding conditioning chambers for 45 min. The dose of morphine and naloxone was selected based on our previous work (Wu et al., 2014). Post-C for the expression of morphine/naloxone-induced CPA in a drug-free state (15 min) was performed on the day 11. The procedure of Post-C was the same as the initial baseline preference assessment. The CPA score was defined as the time spent in the morphine/naloxone-paired chamber divided by the total time spent in both the morphine/naloxone and the saline-paired chambers during CPA testing.

### Experiment Design

#### Experiment 1: Effect of Microinjection of 5-aza into AI and GI on Reconsolidation of Morphine-Associated Withdrawal Memory

Rats were allowed freely access to all compartments for 10 min as memory reactivation on the day after Post-C (Fan et al., 2010; Xue et al., 2012), and DNA methyltransferase inhibitors 5-aza or vehicle was microinjected into AI or GI immediately after the memory reactivation. Four groups of rats (37 rats) were used in the experiment. Two groups of rats were used to determine whether 5-aza microinjected into AI (1 μg/0.5 μl/side, *n* = 7) or GI (1 μg/0.5 μl/side, *n* = 10) impaired the reconsolidation of morphine-associated withdrawal memory. Another two groups of rats were injected with 0.8% acetic acid into AI (0.5 μl/side, *n* = 8) or GI (0.5 μl/side, *n* = 12) as vehicle control. After microinjection, the rats were sent back into their home cages. After 24 h (Post-T1), 7 days (Post-T7) and 14 days (Post-T14), the rats were placed into the CPA apparatus for 15 min to assess the effect of 5-aza on morphine-naloxone induced CPA.

#### Experiment 2: Effect of Microinjection of 5-aza into BLA and CeA on Reconsolidation of Morphine-Associated Withdrawal Memory

The procedures were conducted similarly with experiment 1. Another four groups of rats (34 rats) were used in experiment 2. Two groups of rats received 0.8% acetic acid microinjection into BLA (0.5 μl/side, *n* = 9) or CeA (0.5 μl/side, *n* = 9) as vehicle control groups, the other two groups of rats received 5-aza microinjected into BLA (1 μg/0.5 μl/side, *n* = 9) or CeA (1 μg/0.5 μl/side, *n* = 7). After 24 h (Post-T1), 7 days (Post-T7) and 14 days (Post-T14), we used a drug-free test to assess the effect of 5-aza on morphine-naloxone induced CPA.

#### Experiment 3: Effect of Microinjection of 5-aza into AI and BLA on Morphine-Associated Withdrawal Memory Without Exposure to Conditioning Chamber

After established morphine-associated aversive memory, rats were microinjected 5-aza or 0.8% acetic acid in AI/BLA on day 12 without memory reactivation. Two groups of rats (13 rats) received 0.8% acetic acid microinjection into AI (0.5 μl/side, *n* = 6) or BLA (0.5 μl/side, *n* = 7) as vehicle control, the other two groups of rats (13 rats) received 5-aza microinjected into AI (1 μg/0.5 μl/side, *n* = 7) or BLA (1 μg/0.5 μl/side, *n* = 6). After 24 h (Post-T1), 7 days (Post-T7) and 14 days (Post-T14), we used a drug-free test to assess the effect of 5-aza on morphine-naloxone induced CPA.

#### Experiment 4: Effect of Microinjection of 5-aza into AI and BLA on Drug-Naive Rats

Following baseline preference test on day 1, rats received twice intraperitoneally injection of saline 4 h apart and then were confined to one of the larger chambers for 45 min for eight consecutive days. Each rat was confined to one larger chamber and the other larger chamber on alternating days. Post-C was performed in a drug-free test (15 min). Two groups of rats (8 rats) received 5-aza microinjection into AI (1 μg/0.5 μl/side, *n* = 4) or BLA (1 μg/0.5 μl/side, *n* = 4).We used a drug-free test to assess the effect of 5-aza on drug- naive rats after 24 h (Post-T1).

### Cannula Verification

At the end of experiments, the rats were anesthetized with sodium pentobarbital (75 mg/kg) and transcardially perfused. Cannula placements were assessed using Nissl staining with a section thickness of 40 μm under light microscopy. Rats with misplaced cannulas were excluded from statistical analysis.

### Statistical Analysis

The statistical analysis was performed using two-way repeated measures analysis of variance (ANOVA), with CPA score as the dependent factor. *Post hoc* analyses of significant effects were performed using the Bonferroni test. The preference score was analyzed by paired samples *t*-test to assess whether the rats formed conditioned place preference after saline conditioning. Values of *p* < 0.05 were considered statistical significant. All data was expressed as mean ± SEM and analyzed with Prism 5 Software.

## Results

### Cannula Verification

**Figure 1:**
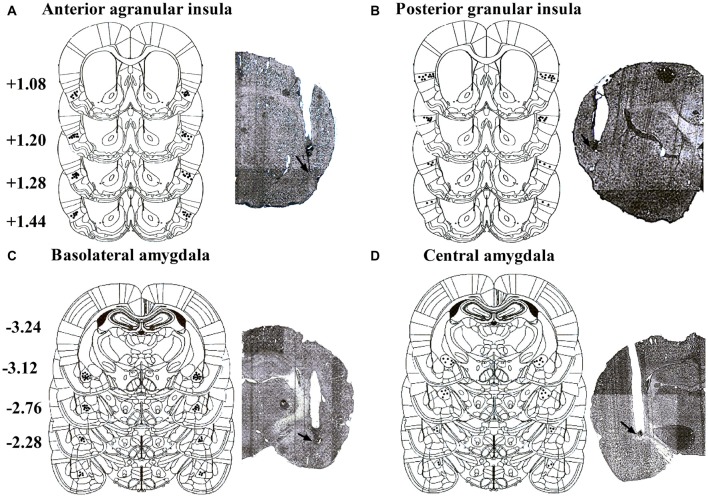
**Schematic illustrations and representative photomicrographs of the intracranial cannula infusion sites in the agranular insular (AI; A), granular insular (GI; B), basolateral amygdala (BLA; C) and central amygdala (CeA; D).** Numbers besides the sections indicate anteroposterior distance from bregma in millimeters. Data are reconstructed from Paxinos and Watson (2007).

Placements of infusion needle tips targeted at AI, GI, BLA and CeA were examined by postmortem histological verification. Twenty-three rats were removed because their placements were outside the scopes. Schematic illustrations and representative photomicrographs of the intracranial cannula infusion sites were presented in Figure 1.

#### Microinjection of 5-aza into the AI Disrupted Reconsolidation of Morphine-Associated CPA

**Figure 2:**
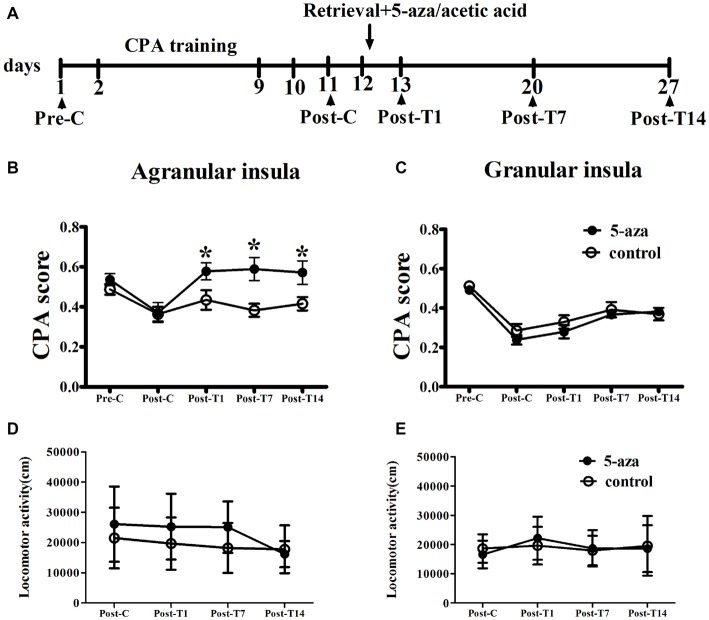
**Effects of microinjection of 5-aza-2′-deoxycytidine (5-aza) into AI and GI on the reconsolidation of conditioned place aversion (CPA). (A)** Timeline of experimental procedure **(B)** 5-aza injection into AI blocked the reconsolidation of CPA. **(C)** Injection of 5-aza into GI did not disrupt the reconsolidation of CPA. **(D,E)** The locomotor activity of all groups in experiment 1. Data are presented as mean ± SEM. *p < 0.05, compared to control at the same time point.

As shown in Figure 2, with treatment (5-aza or 0.8% acetic acid) as the between-subjects factor and test point (Pre-C and Post-C) as the within-subjects factor, ANOVA revealed a significant main effect of test point (*F*_(1,13)_ = 44.37, *p* < 0.001), indicating that the rats in AI groups acquired CPA. Moreover, there was no significant main effect of treatment (*F*_(1,13)_ = 0.01, *p* > 0.05), suggesting that no group differences before 5-aza administration. To examine the effects of 5-aza on the reconsolidation of morphine-withdrawal memories, the rats received 5-aza or 0.8% acetic acid microinjection into the AI immediately after the retrieval test on the day after Post-C. The two-way repeated measures ANOVA conducted on CPA scores, with treatment (5-aza or 0.8% acetic acid) as the between-subjects factor and test point (Post-C, Post-T1, Post-T7 and Post-T14) as the within-subjects factor. ANOVA revealed significant effects of treatment (*F*_(1,13)_ = 4.70, *p* < 0.05) and test point (*F*_(3,39)_ = 5.56, *p* < 0.05) and treatment × test point interaction (*F*_(3,39)_ = 3.65, *p* < 0.05) in the AI. The *post hoc* analyses confirmed that CPA scores significantly decreased in the group of rats that received 5-aza infusion into the AI (*p* < 0.05; Figure 2B). Moreover, as depicted in Figure 2D, 5-aza injection into AI had no effect on the locomotor activity of rats (*p* > 0.05).

In GI groups, two-way ANOVA was performed with treatment (5-aza or 0.8% acetic acid) as the between-subjects factor and test point (Pre-C and Post-C) as the within-subjects factor. ANOVA revealed a significant main effect of test point (*F*_(1,20)_ = 125.70, *p* < 0.001), indicating that the rats in GI groups acquired CPA. Moreover, there was no significant main effect of treatment (*F*_(1,20)_ = 1.25, *p* > 0.05), suggesting that no group differences before 5-aza administration. The effect of 5-aza on reconsolidation of CPA in GI was summarized in Figure 2C. With treatment (5-aza or 0.8% acetic acid) as the between-subjects factor and test point (Post-C, Post-T1, Post-T7 and Post-T14) as the within-subjects factor, the ANOVA revealed no significant main effect of treatment or treatment × test point interaction in the GI (*p* > 0.1). As depicted in Figure 2E, 5-aza injection into GI had no effect on the locomotor activity of rats (*p* > 0.05). The results suggested that blocking DNMTs in AI but not GI with 5-aza after memory reactivation disrupted the reconsolidation of established CPA.

#### Microinjection of 5-aza into BLA Attenuated Retention of Morphine-Associated CPA

**Figure 3:**
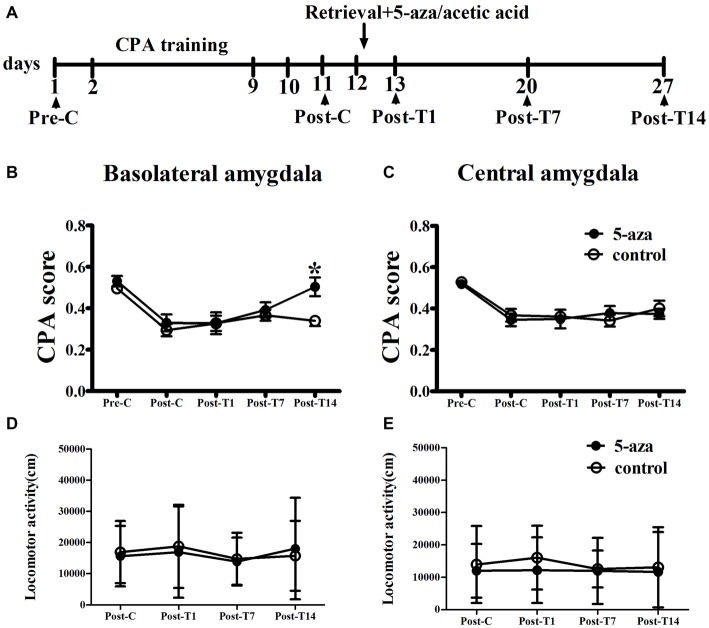
**Effects of microinjection of 5-aza into BLA and CeA on the reconsolidation of CPA. (A)** Timeline of experimental procedure **(B)** 5-aza injection into BLA attenuated the reconsolidation of CPA. **(C)** Injection of 5-aza into CeA did not disrupt the reconsolidation of CPA. **(D,E)** The locomotor activity of all groups in experiment 2. Data are presented as mean ± SEM. *p < 0.05, compared to control at the same time point.

As shown in Figure 3, two-way ANOVA was conducted with treatment (5-aza or 0.8% acetic acid) as the between-subjects factor and test point (Pre-C and Post-C) as the within-subjects factor. ANOVA revealed a significant main effect of test point (*F*_(1,16)_ = 47.82, *p* < 0.001), indicating that the rats in BLA groups acquired CPA. Further, there was no significant effect of treatment (*F*_(1,16)_ = 1.24, *p* > 0.05) suggesting that no group differences before 5-aza administration. Then 5-aza was administered into the BLA and reconsolidation of CPA was evaluated as described in “Materials and Methods” in Section. The two-way repeated measures ANOVA, with treatment (5-aza or 0.8% acetic acid) as the between-subjects factor and test point (Post-C, Post-T1, Post-T7 and Post-T14) as the within-subjects factor, showed significant effect of treatment × test point interaction (*F*_(3,48)_ = 3.73, *p* < 0.05). The *post hoc* analyses confirmed that CPA scores significantly decreased at Post-T14 (*p* < 0.05, compared to control) in the group of rats that received 5-aza infusion (Figure 3B). These results indicated that the effect of 5-aza injection into the BLA on withdrawal memory reconsolidation might be extremely weak.

In CeA groups, the two-way ANOVA was conducted with treatment (5-aza or 0.8% acetic acid) as the between-subjects factor and test point (Pre-C and Post-C) as the within-subjects factor. ANOVA revealed a significant effect of test point (*F*_(1,14)_ = 60.27, *p* < 0.001), indicating that the rats in BLA groups acquired CPA. Moreover, there was no significant effect of treatment (*F*_(1,14)_ = 0.26, *p* > 0.05), suggesting that no group differences before 5-aza administration. The effect of 5-aza on reconsolidation of CPA in CeA was summarized in Figure 3C. The two-way repeated measures ANOVA, with treatment (5-aza or 0.8% acetic acid) as the between-subjects factor and test point (Post-C, Post-T1, Post-T7 and Post-T14) as the within-subjects factor, revealed no significant effect of treatment or treatment × test point interaction in the CeA (*p* > 0.1). As depicted in Figures 3D,E, the ANOVA demonstrated that there was no significant main effect of “5-aza treatment” on the locomotor activity in BLA groups (*p* > 0.05) or CeA groups (*p* > 0.05).

#### Microinjection of 5-aza into AI and BLA Without Memory Reactivation had no Effect on Morphine-Associated CPA

**Figure 4:**
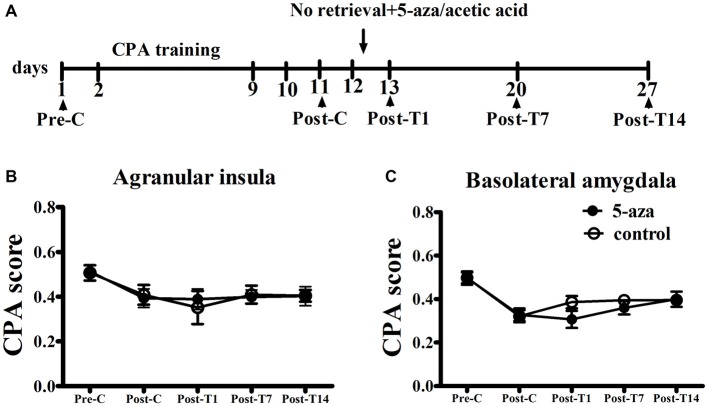
**Effects of microinjection of 5-aza into AI and BLA on morphine-withdrawal memories without exposure to conditioning chamber. (A)** Timeline of experimental procedure **(B)** 5-aza injection into AI without memory reactivation had no effect on CPA. **(C)** Injection of 5-aza into BLA without memory retrieval had no effect on CPA. Data are presented as mean ± SEM.

As shown in Figure 4, two-way ANOVA was conducted respectively in AI groups and in BLA groups with treatment (5-aza or 0.8% acetic acid) as the between-subjects factor and test point (Pre-C and Post-C) as the within-subjects factor. ANOVA revealed that significant main effect of test point in AI groups (*F*_(1, 11)_ = 21.45, *p* < 0.001) and in BLA groups (*F*_(1,11)_ = 94.55, *p* < 0.001), indicating that all groups acquired CPA. There were no significant main effect of treatment in AI (*F*_(1,11)_ = 0.01, *p* > 0.05) or BLA (*F*_(1,11)_ = 0.01, *p* > 0.05) groups, suggesting that no group differences before 5-aza administration. To examine the effect of 5-aza injection without memory reactivation on morphine-withdrawal memories, the rats received 5-aza or 0.8% acetic acid microinjection into the AI or BLA on the day after Post-C (day 12). The two-way repeated measures ANOVA was conducted on CPA scores, with treatment (5-aza or 0.8% acetic acid) as the between-subjects factor and test point (Post-C, Post-T1, Post-T7 and Post-T14) as the within-subjects factor. ANOVA showed no significant main effect of treatment (*F*_(1,11)_ = 0.67, *p* > 0.05) or test point × treatment interaction in BLA groups (*F*_(3,33)_ = 1.77, *p* > 0.05). Similarly, there was no significant main effect of treatment (*F*_(1,11)_ = 0.003, *p* > 0.05) or test point × treatment interaction in AI groups (*F*_(3,33)_ = 0.33, *p* > 0.05). These results suggested that blocking DNMTs in AI or BLA with 5-aza without memory reactivation had no effect on established CPA.

#### Microinjection of 5-aza into AI and BLA had no Effect on Drug-naïve Rats

**Figure 5:**
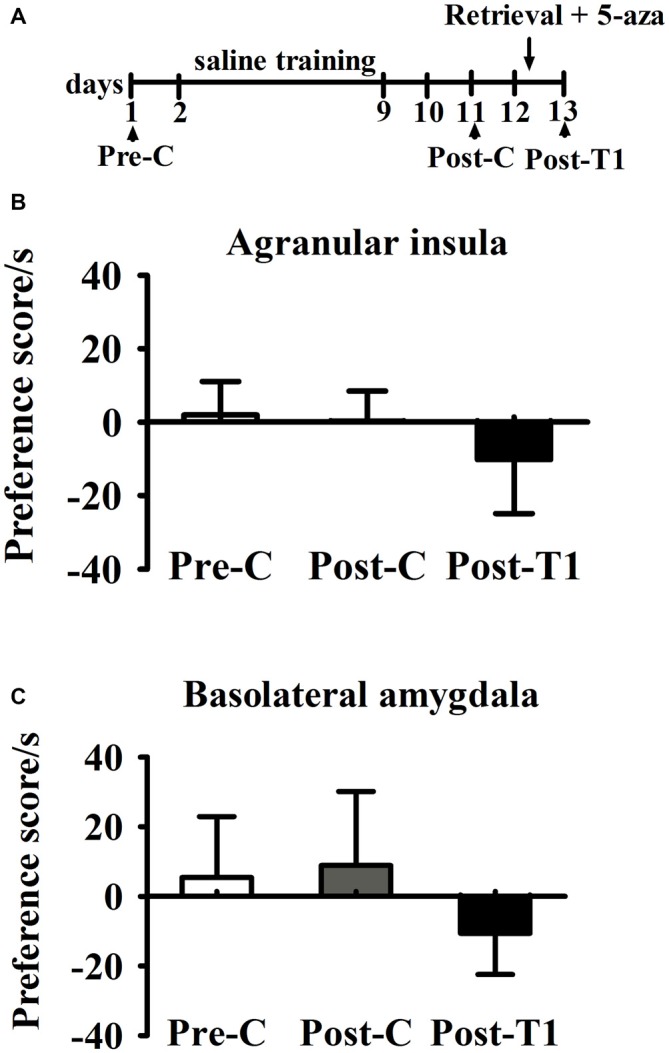
**Effects of microinjection of 5-aza into AI and BLA on drug-free rats. (A)** Timeline of experimental procedure **(B)** 5-aza injection into AI had no effect on CPA. **(C)** Injection of 5-aza into BLA had no effect on CPA. Data are presented as mean ± SEM.

As shown in Figure 5, paired samples *t*-test showed that there was no significant difference between Pre-C and Post-C in AI group (*t*_(3)_ = 2.93, *p* = 0.60) or BLA group (*t*_(3)_ = 1.08, *p* = 0.36), indicating that the rats failed to acquire conditioned place preference after saline training. Then 5-aza was administered into the AI or BLA after 10 min test, and paired samples *t*-test showed no significant effect between Post-C and Post-T1 in AI group (*t*_(3)_ = 0.23, *p* = 0.83) or BLA group (*t*_(3)_ = 0.15, *p* = 0.89). These results suggested that microinjection of 5-aza into the AI or BLA had no effect on the preference/aversion of drug-naive rats.

## Discussion

In the present study, we investigated region-specific effects of DNMTs inhibition in the insular cortex and amygdala on the reconsolidation of morphine-withdrawal memory. We showed that microinjection of 5-aza into the AI, but not GI, disrupted the reconsolidation of morphine-withdrawal memory, and this effect lasted for least 2 weeks. Inhibition of DNMTs activity in the BLA but not CeA, weakened the established CPA at 14 days after retrieval test. Additionally, these effects of DNMTs inhibition required that the memories be actively retrieved. Lastly, our results showed that 5-aza had no effect on locomotor activity and did not induce a preference or aversion on its own. These results suggest that DNA methylation in the AI and BLA are required for the reconsolidation of opiate-withdrawal memory.

The amygdala and insular cortex, especially the BLA and AI, are important components of neural circuits underlying drug-related memory reconsolidation (Hellemans et al., 2006; Contreras et al., 2012). Studies positively proposed that the BLA is a critical brain region for reconsolidation and storage of CS-withdrawal associations (Gale et al., 2004; Agren et al., 2012). Some researches revealed that N-Methyl-D-aspartate (NMDA) Receptors, Zif268, protein kinase Mζ (PKMζ), cyclin-dependent kinase 5 (Cdk5), and transcription factor NF-κB in the BLA serve to stabilize memory reconsolidation (Hellemans et al., 2006; Milton et al., 2008; Li et al., 2010; He et al., 2011; Si et al., 2012). The molecular mechanism of reconsolidation in the AI remains unknown. Our results showed that microinjection of 5-aza into the AI disrupted the reconsolidation of morphine-withdrawal memory, while microinjection of 5-aza into the BLA weakened the subsequent CPA expression. These findings suggested that DNMTs activity in the BLA and AI are involved but play distinct roles in the reconsolidation of opiate-withdrawal memories. Moreover, we found that microinjection of 5-aza into the GI and CeA did not affect the reconsolidation. In combination with previous findings, the GI seem to be the key sub-regions to receive and encode primary aversive information (Allen et al., 1991), but not involved in aversive memory reconsolidation. Nevertheless, the reconsolidation of morphine-associated aversive memory in the CeA might not underlie DNA methylation modification. In addition, our results showed that the memory impairment induced by DNMTs inhibition in the AI and BLA was dependent on memory recall, because the memory impairment was not observed when DNMTs inhibition occurred in the absence of memory retrieval. The necessity of DNMTs inhibition for reconsolidation might be specific to the withdrawal memories, because 5-aza could not induce preference or aversion for saline conditioned rats. Collectively, our findings suggested that the DNMTs activity in the AI and BLA are critical for the reconsolidation process.

Accumulated evidence demonstrated that DNA methylation played an important role in the reconsolidation of fear memories (Maddox and Schafe, 2011; Maddox et al., 2014). The highly expression of three subtypes of DNMTs (DNMT1, DNMT3a, DNMT3b) in adulthood (Anier et al., 2010; LaPlant et al., 2010) suggested that dynamic regulation of DNA methylation might be critical for neuronal function, including synaptic plasticity and memory modulation. Studies showed that DNMTs activity in the LA is critical not only for the reconsolidation of fear memory (Maddox and Schafe, 2011), but also for the reconsolidation-associated neural plasticity (Maddox et al., 2014). In the present study, we found blockade of DNMTs activity in the AI and BLA interfered with the reconsolidation of morphine-associated aversive memory in different manners. Our findings broaden the scope of research on DNA methylation, particularly in reconsolidation of opiate-withdrawal memories.

How DNA methylation modulates the reconsolidation of withdrawal memory? We presumed that DNMTs transiently increase the DNA methylation of downstream target genes, which ultimately influences the reconsolidation. The DNMTs inhibition is involved in promoting expression of memory suppressing genes, such as protein phosphatase 1 (PP1), which is an important molecule in the consolidation of fear memory (Miller and Sweatt, 2007) and addiction memory (Zhang et al., 2014). Meanwhile, PP1 is regulated by DNA methylation (Anier et al., 2010). Thus, our results suggested that DNMTs inhibition in the AI and BLA impaired reconsolidation of morphine-withdrawal memory, which might act through promoting the expression of memory suppressor genes. Another explanation would be the interaction between DNA methylation and histone acetylation. Several researches have indicate that DNA methylation and histone acetylation work in concert to regulate memory (Miller et al., 2008; Maddox and Schafe, 2011). Effects of DNMTs inhibition on the fear memory reconsolidation could be reversed by an histone deacetylase (HDAC) inhibitor (Maddox and Schafe, 2011). It seems likely that HDAC inhibitor overcomes the memory deficit produced by 5-aza by increasing the transcriptional activity of memory promoter genes, whose transcription is upregulated by HDAC inhibition (Vecsey et al., 2007). These genes may then overcome the memory suppressing effects of genes that were made aberrantly active by the DNMT inhibition, ultimately resulting in normal memory formation. Therefore, DNMTs inhibition might impair the reconsolidation of withdrawal memory via regulation of histone acetylation in the AI and BLA. Collectively, the DNA methylation mechanism underlying the reconsolidation of aversive memory is complex, and a series of studies are needed to be done in the future.

We also found distinctive effects of microinjection of 5-aza into the AI and BLA on the reconsolidation of morphine-withdrawal memory. Microinjection of 5-aza into the BLA resulted in the instability of morphine-withdrawal memories, which could not maintain 14 days after microinjection of 5-aza, whereas microinjection of 5-aza into the AI disrupted the reconsolidation so that memory could not maintain 24 h thereafter. One explanation for the distinctive effects is that DNA methylation in the AI and BLA occurs at different points of time during the reconsolidation process. Evidence described the time-dependent reorganization of brain circuitry underlying long-term memory storage. For example, the changes of metabolic activities in the hippocampus is prior to that in the neocortex in retention of a spatial discrimination task (Bontempi et al., 1999). This raised the intriguing idea that the points of time when DNA methylation happens in the AI and BLA are varied. Based on our findings, the DNA methylation in the AI might be upregulated immediately after the memories retrieval, while the DNA methylation upregulation in the BLA might be delayed. Thus, one possibility of our results is that 5-aza took effect immediately after memory retrieval in AI, and strongly inhibited DNA methylation process. Whereas, most of 5-aza might be metabolized in the BLA when the DNA methylation upregulated, resulting in low dose of 5-aza and its weak effect on withdrawal memory reconsolidation. Similar results also showed that relatively low dose of protein synthesis inhibitors have no effect on the reconsolidation or just weaken the process (Yu et al., 2013). Another explanation would be that microinjection of 5-aza into the AI might impair the reconsolidation, whereas memory deficits in BLA could be a result of enhanced extinction learning. Obviously, additional experiments are required to further confirm the above hypothesis.

The present findings, for the first time, showed the pharmacological evidence that 5-aza contributes to the reconsolidation of conditioned opiate-withdrawal memory. We also found the distinctive effects of 5-aza injection into the AI and BLA on the reconsolidation of opiate-associated aversive memory.

## Author Contributions

PL designed the experiments, performed the experiments, analyzed data and wrote article; JJZ and ML designed the experiments and revised the article; NS designed research, wrote article and had primary responsibility for final content.

## Conflict of Interest Statement

The authors declare that the research was conducted in the absence of any commercial or financial relationships that could be construed as a potential conflict of interest.
